# Narcolepsy: Pathophysiology and Neuropsychological Changes

**DOI:** 10.1155/2003/323060

**Published:** 2003-01-01

**Authors:** Angela Naumann, Irene Daum

**Affiliations:** Department of NeuropsychologyInstitute of Cognitive NeuroscienceRuhr-University of BochumGermany

## Abstract

Narcolepsy is now recognized as a distinctive disorder with specific pathophysiology and neurochemical abnormalities. Findings on the role of the neuropeptide hypocretin are opening new avenues of research and new strategies for therapy. Recently, neuropsychological and electrophysiological studies have provided evidence for reduced memory performance on standard memory tests in addition to subjective complaints of forgetfulness which may be related to changes in attentional processing. Further studies are, however, necessary to clarify the neuropsychological profile in narcolepsy. This review focuses on the recent advances in understanding narcolepsy.

## 1. Historical overview

Early descriptions of unusual excessive sleepiness appeared as early as 1829 and 1836 in publications by Schindler and Bright [35,72]. However, the first clear case of the disorder that was later called narcolepsy was described in 1877 by Westphal in his article “Eigenthümliche mit Einschlafen verbundene Anfälle” (peculiar attacks associated with falling asleep). The patient reported sudden myasthenia, sleep attacks arising without loss of consciousness and disrupted sleep patterns during the night [134]. A few years later in 1880, Gelineau [39] introduced the term narcolepsy (Grecian $\alpha pk\eta$: “convulsion” and $\lambda\varepsilon\pi\sigma\iota\sigma$: “receive”) to define pathological conditions characterized by unexpected short sleep episodes as well as spontaneous muscle tone loss which occurred in conjunction with emotional events. Later Hennenberg [51] introduced the term cataplexy for the description of sudden loss of muscle tone, and Redlich [108] used the term “affektiver Tonusverlust” (affective loss of tone) to describe the same symptom. Hypnagogic hallucinations and sleep paralysis were assigned to the narcoleptic syndrome in the 1920ies [1,73,135]. Together with excessive sleepiness and cataplexy, these symptoms constituted the *narcoleptic tetrad* [136]. During the first international conference on narcolepsy, disrupted night sleep was added as the fifth characteristic symptom [46].

Of special importance was the discovery of sleep onset REM Periods (SOREM) in narcoleptic patients in 1960 [133]. This finding led to the suggestion that cataplexy, hypnagogic hallucinations as well as sleep paralysis might be due to changes in REM sleep mechanisms [107,126]. The discovery of narcolepsy in dogs and its genetic basis in the 1970s [33,67] and the observation of the role of the hypocretin system [75,94] are regarded as the beginning of a new era of narcolepsy research.

## 2. Clinical symptoms and diagnostic criteria

Narcolepsy can be clearly distinguished from disorders that are also characterized by excessive sleepiness and nocturnal sleep disturbances, such as sleep apnoea or restless leg syndrome by criteria set by the International Classification for Sleep Disorders [62]. Despite recent criticism, the diagnosis of narcolepsy based on the occurrence of excessive sleepiness and cataplectic attacks alone is still accepted [43,89,112]. Although sleepiness can be assessed with subjective

ISSN 0953-4180/03/$8.00 © 2003 – IOS Press. All rights reserved scales (e.g. Stanford Sleepiness Scale, 55) or electrophysiological recordings, the Multiple Sleep Latency Test (MSLT) is the most useful assessment technique [18]. Polysomnography recordings are carried out at two-hour intervals throughout the day, and a mean sleep latency of less than five minutes and at least two REM periods sleep onset of sleep are considered as pathological, while latencies between five and ten minutes require further assessment [89]. An alternative to the MSLT is the "Maintenance of Wakefulness Test (MWT)", in which the patient sits in a darkened room and is to stay awake [50].

### *2.1. Excessive sleepiness*

The most important symptom of narcolepsy is excessive sleepiness as indicated by two or more brief sleep attacks during daytime [48]. These short sleep episodes manifest themselves unintentionally and under the most unusual conditions such as reading, working, eating and even when driving. The episodes can last for a few seconds up to 30 minutes [37], and rarely exceed more than one hour [58]. The fatigue level fluctuates over the day, but follows a stereotyped, individual pattern in most cases. In addition, automatic behaviours may occur in 8 to 40% of patients, with simple repetitive activities and meaningless speech and later amnesia for the episode [25,37,114]. Behaviours such as continuing to walk during an attack, writing off the edge of a page or brief lapses in speech are common examples.

### *2.2. Cataplexy*

Another frequent symptom – the cataplectic attack – is defined as a sudden reduction or loss of skeletal muscle tone during full consciousness [46,58]. Severe attacks can cause complete paralysis except for the eye and respiratory muscles. More commonly, the attacks affect individual body regions. Most attacks are triggered by excitement or strong emotions like laughter, surprise, anger or fear. The frequency of the attacks ranges from more than 10 attacks a day to less than one a month [38]. Brief attacks are common and most last only a few minutes [46,58]. Longer durations usually represent several consecutive attacks or REM sleep onset.

### *2.3. Sleep paralysis and hallucinations*

Sleep paralysis is characterized by a complete inability to move when falling asleep (hypnagogic) or waking up (hypnopompic). The patients are subjectively fully awake, but incapable of movement. Sleep paralysis resembles cataplectic attacks, but lasts longer (up to 10 min), occurs less frequently and does not usually involve emotional triggers. In more than half of the patients, it is accompanied by vigorous hallucinations [10,58,97,137], which may also occur independently. Hallucinations are usually visual, less frequently in the auditory or tactile domains, and olfactory or gustatory hallucinations are exceptional. Approximately 20–30% of all narcoleptic patients experience all four symptoms of the narcoleptic tetrad [136].

### *2.4. Fragmented nocturnal sleep*

Nocturnal waking periods, which are found in 60–80% of all narcoleptic patients, are usually of short duration and very rarely last several hours [10,25]. Despite fragmented night sleep, the total sleep time seems to be normal in narcolepsy. There is also no evidence for altered non-REM sleep (NREM) [125], and the frequencies of nocturnal waking and daytime sleep attacks appear to be unrelated [17,69].

### *2.5. Other symptoms*

In addition to the typical symptoms described above, narcoleptics frequently experience vivid and frightening dreams, increased body movements and periodic leg movements in NREM and REM sleep [10,25]. Many patients are overweight, with body mass index (BMI) scores 10–20% over control scores [25,68,118], which has been attributed to increased inactivity due to permanent tiredness [80] and abnormal metabolic rates [68,118]. Sensory and cognitive problems, such as double vision, deafness, headache, and concentration and memory deficits have also been reported, as well as depression, panic attacks and social problems [9,37].

## 3. Epidemiology

The prevalence of narcolepsy ranges from 20 to 60 cases per 100,000 [6,28,61,84,114]. The highest rate is found in Japan [57,84], the lowest in Israel [71]. The large range may be related to differences in diagnostic criteria. Women and men are affected equally. The first symptoms manifest themselves between 15 and 35 years of age in over 80% of cases [19,25,98,137]. Life expectancy is normal. The symptoms appear gradually, with excessive sleepiness and spontaneous sleep attacks as typical initial symptoms. While partial remissions have been observed, complete remissions have not been reported.

## 4. Pathology

The clinical features of narcolepsy have been described in terms of a "loss of state boundary control" [16]. This is characterised by an inability to maintain a given sleep/awake status over a longer time period and the fact that phenomena that normally occur in conjunction with sleep manifest themselves outside of their usual context. Based on these assumptions, several explanations for the pathophysiology of narcolepsy have been proposed.

### *4.1. Pathological characteristics of narcoleptic sleep*

While REM (rapid eye movement) sleep is normally first observed after approximately ninety minutes, REM at sleep onset or within 10 minutes thereafter (SOREM) is the most characteristic feature of narcolepsy [8,29, 133]. A similar pattern is found during brief daytime sleep attacks, in which REM sleep characteristics also occur at the beginning [54,99]. This finding led to the hypothesis that narcolepsy is caused by an imbalance of the physiological processes that control transitions between waking and sleep states [6,16]. Dissociated manifestations of REM sleep that intrude into wakefulness could be an explanation for sleep paralysis and cataplexy [29,45,54,107,133]. Sleep paralysis only occurs during periods of SOREM, and REM sleep often follows long attacks of cataplexy. Additionally, atonia observed during cataplectic attacks and sleep paralysis appears to be physiologically similar to atonia that usually accompanies normal REM sleep [54]. The fact that hypnagogic hallucinations occur only during periods of SOREM and their similarity to normal REM dream experiences also imply modified REM sleep mechanisms [74]. In summary, the pathophysiological characteristics indicate that narcoleptic symptoms are probably due to changes of physiological sleep control systems [120].

### *4.2. Neurotransmitter dysfunction in narcolepsy*

Several lines of evidence relate narcolepsy to dysfunction of cholinergic and monoaminergic neurotransmitter mechanisms that are critically involved in the regulation of normal sleep. Drugs which enhance the synaptic availability of noradrenalin inhibit cataplexy in dogs, whereas exacerbation of cataplexy was observed after administration of prazosin, a selective alpha-1 adrenergic receptor blocker [5,70,81]. Clomipramine, a serotonergic uptake inhibitor, reduced the number of cataplectic attacks which was interpreted in terms of a primary serotonergic dysfunction or a secondary mediation via the noradrenergic system [70]. Altered alpha-1 and alpha-2 receptor binding in the amygdala, globus pallidus, putamen and nucleus caudatus, as well as increased rates of specific monoaminergic metabolites are also in line with altered monoaminergic functioning in narcolepsy [4,66].

The cholinergic and the dopaminergic systems have also been implicated in narcolepsy symptoms. Activation of the cholinergic system via M2 stimulation increases cataplexy, and M2 as well as D2/D3 receptor abnormalities in the pons have been documented in animal models [12,56,65,78,91–93,95,109]. The CSF concentration of dopamine and its metabolites was found to be decreased in narcoleptic patients [30,88]; the results for D1- and D2-receptor binding in the basal ganglia and the amygdala have been inconsistent [7,60, 64,66,110].

## 5. Genetic factors

Estimates of the incidence of narcolepsy among first degree relatives range from 2 to 50% [11,44,98]. The influence of genetic factors on narcolepsy onset is widely acknowledged, but the mechanisms are still largely unknown. The concordance rates for monozygotic twins are 25 to 31% [84] which indicates a complex etiology, including genetic factors affecting the expression of narcolepsy and environmental factors.

In support of a genetic basis of narcolepsy, recent studies reported that specific subtypes of human leucocyte antigen, HLA DQB1*0606 and HLA-DR2, are found in nearly 100% of all narcoleptics [58,84]. The HLA complex covers six genes located on chromosome six. Since the HLA subtypes in question are present in 20 to 30% of the population and since these rates far exceed the prevalence of narcolepsy, they do not represent a primary cause, but may affect the expression of other factors.

Although most diseases associated with specific HLA alleles are autoimmune illnesses, there is so far no clear evidence of autoimmune reactions in narcolepsy [34,53]. It has been suggested, however, that an autoimmune reaction may lead to a transient limited neuronal cell loss, which is in line with recent findings on the hypocretin system [83].

### 5.1. The hypocretin system

There are two hypocretin neuropeptides, hypocretin 1 (hcrt-1 or orexin-A) and hypocretin 2 (hcrt-2 or orexin-B), which are generated from a single precursor (prehypocretin) and synthesized by a small number of neurons in the lateral hypothalamus [100,127]. Hypocretin-positive fibers and terminals and the two hypocretin receptors (hcrtr-1 and hcrtr-2) are widely distributed in the central nervous system, including the basal forebrain, the amygdala, the locus coeruleus, and the raphe nuclei as well as the hypothalamus and the thalamus [22,26,100,128]. Because of their amino acid structure and their localization, the hypocretin peptides are regarded as neurotransmitters [131].

Investigations in dogs, which suffer from sporadic autosomal recessive narcolepsy provided evidence of a hcrtr-2 mutation [75]. Three different hcrtr-2 mutations have been described to date, all three being associated with a complete loss of function, while hcrt-1 mechanisms were intact [111]. Prehypocretin knockout in mice produced a narcolepsy-like phenotype [21].

Surprisingly, except for one case, there is no evidence of hcrt-1, hcrt-2, hcrtr-1 or hcrtr-2 mutations in humans [101]. However, Nishino and colleagues observed low hcrt-1 levels in the CSF in seven of nine narcoleptic patients, and post-mortem studies indicated a severe reduction of hypocretin-containing neurons in the hypothalamus of narcoleptic patients [94,101,127]. The selective destruction of hypocretin-producing cells may be the result of postnatal degeneration, altered neural development or reduced synthesis and/or release of hypocretin peptides, the hypothesis of postnatal degeneration being currently favoured [127]. Given the close association between HLA subtypes and narcolepsy, it is conceivable that the specific cell loss could be to due to an autoimmune reaction [58,82].

The hypocretin system is known to play a role in the regulation of food intake and energy balance [42, 76], its function in normal sleep regulation is, however, still largely unclear. Hcrt-1 induced an increase in general arousal level, in combination with stereotypic motor activity and enhanced firing rates in the locus coeruleus [47,102]. Injection of hcrt-1 into the locus coeruleus led to a dose-dependent suppression of REM sleep [13], after hcrt-1 injection in the lateral preoptic area of rats [79]. As to the mechanisms leading to narcolepsy symptoms, it is likely that dysfunction in the hypocretin system may affect cholinergic and monoaminergic activity during sleep cycles [85].

## 6. Cognitive changes in narcolepsy

### 6.1. Memory

Sleep state and level of sleepiness are considered to have profound effects on the ability to form new memories and retrieve old memories. Memory consists of several processes: a stimulus registration or acquisition phase of short duration, a consolidation phase from short-term to long-term memory, and retrieval from long-term memory. Scientists have also postulated a number of different memory systems which elaborate long-term memory. Findings of sleep deprivation studies (SD), which investigate the performance of healthy persons when their normal sleep pattern is manipulated, are sometimes used to understand cognitive profiles associated with sleep disorders. However, the memory and sleep literature, while extensive, is complex and somewhat contradictory [77,121,123,132]. For example Harrison and Horne [49] observed that SD significantly impaired performances in short and novel language tasks having a prefrontal focus. Sleep loss appeared to interfere with novel responses and the ability to suppress routine answers. Quigley et al. [106] measured psychomotor and memory functions in eight sleep-deprived healthy volunteers and eight controls, but memory function was not affected. Therefore further investigations are necessary to clarify whether or not increased sleepiness due to sleep deprivation or sleep restriction is differentially disruptive of particular memory processes.

Early reports of cognitive impairment in narcolepsy were mainly based on self-report complaints of individual patients or anecdotic clinical observations. Independent of the degree of sleepiness, sufferers showed problems in following conversations over a longer period of time, omitted words while speaking and complained of forgetfulness [37]. In a study of 46 narcoleptic patients, 47.8% stated that their memory had deteriorated since the onset of symptoms which mainly affected memory for recent information [15]. Comparable problems were only reported by 4.7% of matched controls in the appropriate period of life. These findings were replicated by Broughton et al. [14] in 1981. In a survey of 180 narcolepsy patients from Asia, Europe and North America, 84 patients (46.7%) reported memory impairments, in particular for recently experienced events. In a more recent study Smith et al. [122] observed that 38% of 700 interviewed narcoleptics reported moderate or severe memory problems, 39% had problems with forgetfulness, 40% with concentration and 26% with general learning processes.

Based on these self reports several studies have attempted an objective evaluation of the subjective experiences of narcoleptic patients of diminished memory function. Aguirre, Broughton and Stuss [2] compared the performance of narcoleptics and matched controls on a range of short- and long-term memory tasks, including verbal and non-verbal as well as acoustic and visual procedures. Despite patients' subjective complaints, no significant group differences were found and the authors attributed the subjective complaints to the patients' problems in sustaining alertness during normal everyday activities. These results were confirmed by Rosenberg and Rogers [113], who reported intact performance of narcoleptics on immediate and delayed recall as well as verbal and visual memory. In addition, Smith et al. [122] examined narcoleptic patients regarding global cognitive functions using a range of neuropsychological tests. Although narcoleptic patients had higher self-rated memory problems, these self-perceived difficulties were not reflected in their performance differences on memory tasks. In contrast, Henry, Satz and Heilbronner [52] observed selective cognitive deficits in narcolepsy in response latency, word recall, and estimation of frequency, while there was no distinction in motor speed, vigilance, information processing speed or decision-making accuracy. The impaired performance was attributed to a perceptual-encoding deficit. Such a deficit may diminish the efficiency with which targets are attended to and processed and could underlie problems in memory and complex reaction time associated with narcolepsy. Neither Aguirre, Broughton and Stuss [2] nor Henry, Satz and Heilbronner [52] could detect an influence of antinarcoleptic treatment on cognitive performance, including methylphenidate for sleepiness and sleep attacks as well as antidepressants to suppress cataplexy and other REM-sleep-related symptoms. Although implicit memory aspects have not yet been studied directly, Pollak et al. [103] observed a lack of practice effects across sessions on cognitive and motor tasks (e.g. serial search and verbal reasoning) in narcoleptics, suggesting a potential problem in skill acquisition.

Based on these findings, many neuropsychologists suggested that narcolepsy is not associated with a primary memory impairment and proposed that functional changes in other areas (i.e. attention and concentration) may underlie subjective complaints. Moreover Hood and Bruck [59] argued that an underlying effect of depression generated by narcolepsy may also account in part for the perceived memory difficulties. Factors of increased global anxiety [124], reduced global self-efficacy and more pervasive negative psychosocial functioning in narcolepsy, including diminished educational opportunities [63], had to be considered as potential explanations of diminished self-efficacy in cognitive domains. However, the small number of correlational analyses between psychosocial adjustment to narcolepsy and subjective estimations of cognitive dysfunction do not yet allow a clear conclusion.

### *6.2. Attention*

Today it is generally accepted that attention is not a unitary construct but has several components, including alertness, vigilance, selective and divided attention [104,105]. Especially reduced vigilance– an inability to sustain full alertness during normal activity– is frequently cited as a deficit associated with narcolepsy. Valley and Broughton [130] found that although instructed to stay awake and perform a 1-hour Wilkinson auditory vigilance task, narcoleptic patients performed significantly worse and were significantly less wakeful than controls. Schulz and Wilde-Frenzen [119] measured critical flicker fusion (CFF) at 15-min intervals for 10 hours to assess fluctuations in vigilance and circadian variations in performance. The narcoleptic patients showed increased CFF variability throughout the day, which was present from the beginning of the session. This finding was interpreted as a "waxing and waning" state of alertness. Along similar lines, narcoleptics showed difficulty in maintaining constant vigilance levels during computer-based driving simulation tests that was unrelated to level of tiredness [31,32,40]. In addition, narcoleptic patients experienced more difficulty in maintaining attention than control subjects, as evidenced by significantly more perseveration errors on Strub and Black's List of Letters [113]. Taken together, if tests are grouped retrospectively into those which showed performance deficits and those which did not, a rather consistent pattern appears. Tests that show deficits in narcolepsy tend to be those that are relatively long and repetitious, including for example a 60-min auditory vigilance test, a 10-min choice reaction time task, or a 20-min digit symbol substitution test (DSST) [41,86,129]. In contrast, on shorter, more challenging attention tasks, such as the Paced Auditory Serial Addition Test (PASAT) or Digit Span test, the performance of narcoleptics was generally unimpaired [113,129], although deficits on a brief visual search task have been reported [103]. The accuracy in this task was slightly lower in narcoleptic patients compared to matched controls, which was explained in part by the possibility of micro-sleeps in narcoleptics, defined as sleep episodes of less than 15 seconds.

Overall the published findings strongly suggest that narcoleptic patients suffer from attention deficits, in particular if attention has to be maintained over longer periods of time. Altered sleep regulation mechanisms may possibly induce continuous fluctuations of vigilance in narcolepsy, which in turn allow only sporadic periods of sustained attention and may interfere with cognitive processes as concentration, learning and memory. Further investigations are, however, necessary to understand the mechanisms of attentional control in narcolepsy in more detail.

### *6.3. Executive function*

Executive function has been associated with dissociable mental operations including planning ahead and problem solving, shifting between actions easily, initiating goal-directed behaviour, and regulating attention in order to complete tasks. Deficits in executive control have been classically associated with acquired damage to the prefrontal cortex, but recent findings show that executive dysfunctions should not be assumed to reflect damage specifically to the frontal lobes, as such deficits may also arise from damage to interconnected cortical and subcortical brain structures or from more diffuse brain damage [20,23,36]. Although the study of executive functioning is a relatively new and rapidly growing field of research, the data concerning executive processes in narcolepsy are sparse. Early findings were published by Valley and Broughton in 1981 [129]. They examined narcoleptic patients in a classical working memory task (Digit Span Backwards) and observed no significant performance difference compared to healthy controls. This finding was replicated by Aguirre, Broughton and Stuss [2] as well as Rogers and Rosenberg [113]. In addition, comparisons between narcoleptics and controls were nonsignificant on a verbal fluency test, a complex verbal reasoning task and a working memory task, which examined coding and representation of information in relation to time and place of occurrence [2,52,103]. In contrast, narcoleptics displayed selective cognitive deficits using Sternberg's STM Scanning Task, which is a short- term as well as working memory task [52]. However, because of the small number of studies, the limited data situation, and small sample sizes, as well as the different methodological procedures the evaluation of executive functions in narcolepsy remains difficult. Therefore more studies concerning the functionality of executive processes in narcolepsy would be useful.

### *6.4. Information processing as indicated by event-related potentials*

Although the analysis of event-related potentials (ERPs) has become a useful assessment tool in the neurosciences, electrophysiological studies with narcoleptic patients are rare. In a study by Ollo et al. [96], ERPs were recorded during an "easy" and a "difficult" auditory oddball task. Narcoleptics showed reduced P300 amplitudes when active attention was required, but not during distraction or ignore-conditions, indicating dysfunctional attentional mechanisms. Since sleepiness which as such can lead to an amplitude reduction [24] was not assessed, the potential confounding effect of tiredness cannot be ruled out. This issue is of relevance, since reduced P300 amplitudes, together with normal contingent negative variations (CNV) were observed in conjunction with high scores on the Stanford Sleepiness Scale [3]. Co-existing sleep apnoea which was present in 7 out of 12 patients of the Aguirre and Broughton [3] study, also has to be considered as a potential confounding variable [27,87].

Although P300 latencies were found to be normal in earlier studies [3,96,116], recent investigations using larger samples of narcoleptic patients reported prolonged P300 latencies to visual as well as auditory stimuli, while amplitudes were unimpaired. Changes in P300 latency were unrelated to sleepiness [115,117]. A study by Naumann et al. [90] which focussed on P300 and mismatch negativity (MMN) as indicators of attentional processing revealed increased P300 amplitudes in narcoleptics compared to controls, in particular at frontal recording sites. While control subjects exhibited the typical pattern of larger MMN over the anterior right compared to the left hemisphere, narcoleptics did not show this asymmetry. This pattern was interpreted in terms of changes in cognitive preattentive and attentive processing, possibly in association with altered functioning of the prefrontal cortex in narcolepsy.

## 7. Conclusions

Recently, major advances have been made with respect to knowledge about the pathophysiological basis of narcolepsy. Findings on the dysfunction of several neurotransmitters and the hypocretin system in particular have offered new insights into the mechanisms underlying deficient control of sleep-waking-processes. The issue of the presence and severity of cognitive dysfunction in narcolepsy and its potential basis, is, however still controversial. Subjective complaints of forgetfulness have not been confirmed by studies using standardised memory tests. There is, however, some neuropsychological and electrophysiological evidence of high levels of fluctuations in vigilance and attention. To further elucidate the nature and pattern of cognitive problems in narcolepsy, investigations in larger samples which consider and control for confounding variables such as level of tiredness at testing, medication and co-existing sleep disorders are clearly needed.
